# Impact of the conversation map tools in patients with type 2 diabetes mellitus: A PRISMA-compliant meta-analysis of randomized controlled trials

**DOI:** 10.1097/MD.0000000000004664

**Published:** 2016-10-07

**Authors:** Qing Yang, Ping Fang

**Affiliations:** a Department of Endocrinology; b Out-patient Department, ZhuJiang Hospital of Southern Medical University, Guangzhou, China.

**Keywords:** conversation map, meta-analysis, type 2 diabetes

## Abstract

**Background:**

Diabetes is one of the leading causes of morbidity and mortality worldwide, and type 2 diabetes is the most common type accounting for 90% of all diabetes cases. Health education is considered as the first choice to control blood glucose levels. We conducted a meta-analysis to assess the effect of health education tool “Conversation Map” in diabetes patients to control blood glucose.

**Methods:**

We searched PubMed, Embase, Cochrane Central Register of Controlled Trials, China Knowledge Resource Integrated Database, and China Science Periodical Database up to December, 2015. We assessed the results using the inverse variance method to pool diabetes relative indicators, and assessed the heterogeneity of the results using I-square.

**Results:**

We collected 22 trials in our meta-analysis, which included 3360 patients. The results showed that the fasting blood-glucose level was significantly reduced in the type 2 diabetes group patients educated with “Conversation Map” when compared to their respective control groups (weighted mean difference [WMD]: −2.23, 95% confidence interval [CI]: −2.70 to −1.76, *P* < 0.001). Also a significant reduction of 2-hour postprandial blood glucose (WMD: −1.59, 95% CI: −2.27 to −0.92, *P* < 0.001) and Hemoglobin A1C levels (WMD: −0.63, 95% CI: −1.08 to −0.17, *P* < 0.001) was also observed when compared to the control groups.

**Conclusion:**

Conversation Map is an effective health education tool for type 2 diabetes, and significantly reduced patients’ blood glucose related index.

## 1 Introduction

The International Diabetes Federation has published a new interactive health education tool for diabetes which is known as “Conversation Map,” and is composed of pictures, dialogue cards, and guidelines. The education model is based on dialogues without any pressure to encourage patients in developing their knowledge to accept diabetes, change their behaviors of daily life, and improve their capacity for self-management. It has been applied to type 2 diabetes patients and has shown promising results. Previous study have proven that “Conversation Map” can control and delay the onset of diabetes and its complications, and have shown better results when compared to other types of education.^[1]^ Therefore, “Conversation Map” has been more recently considered as the first choice in diabetes health education. Recently, numerous studies have evaluated the effect of “Conversation Map” in patients with type 2 diabetes have already completed. Although there have been a significant increase in the number of clinical trials using “Conversation Map,” some trials suggested the effects of “Conversation Map” education in diabetes patients which are still unclear. To better understand any potential impact of “Conversation Map” in patients with type 2 diabetes, data from these studies needs to be evaluated and combined with data from other countries. In this study, we systematically analyzed the effect of “Conversation Map” for diabetes patients, and provide a better clinical guideline for diabetes education.

## 2 Methods

### 2.1 Data sources, search strategies, and study selection

We searched PubMed, Embase, Cochrane Central Register of Controlled Trials, China Knowledge Resource Integrated Database, and China Science Periodical Database using the following keywords:

“Conversation map,” “diabetes,” and “random.” We did not apply any language restrictions and included all relevant articles up to December 2015. We also searched the reference lists of identified trials.

Two reviewers independently identified the eligible reports. Discrepancies were resolved through group discussion. Eligibility criteria included the following requirements: type 2 diabetes patients for treatment; randomized controlled trials; and outcome included at least one blood glucose test. The exclusion criteria included the following: duplicate articles; unrelated research; conference papers; and unclear results. This systematic review adhered to the PRISMA (Preferred Reporting Items for Systematic Reviews and Meta-analyses) Statement and Checklist.^[2]^ Ethics approval was not necessary for this study, as only deidentified pooled data from individual studies were analyzed.

### 2.2 Data extraction and quality assessment

Two authors compiled data using a predefined information sheet. The following data types were extracted from the included articles: author, year, country, sample size, mean age, glycated hemoglobin (HbA1c) levels, fasting blood glucose (FBG) levels, 2 hours postprandial blood glucose levels, Intervention of Diabetes Conversation Map group data, control group data, outcomes, and follow-up results. Two reviewers independently conducted the risk of design bias assessments on the included studies using the 5-points Jadad Scale,^[3]^ and those with 3 to 5 points which represents high-quality trials. The following outcome parameters were quantitatively evaluated in this review: HbA1c, FBG, and 2 hours postprandial blood glucose.

### 2.3 Statistical analysis

We used the inverse variance method to pool continuous data and the results were presented as weighted mean difference (WMD) with 95% confidence intervals (CIs). The I^2^ statistics were calculated to evaluate the extent of variability which was attributed to statistical heterogeneity between trials.^[4]^ In the absence of statistical heterogeneity (I^2^ < 50%), we used a fixed-effect model, a random-effect model was used otherwise.^[5]^ Predefined subgroup analyses were performed on country, patients’ mean age, and intervention time. We assessed for publication bias by visually examining the funnel plots and using the Begg-Mazumdar^[6]^ and Egger tests.^[7,8]^ A nonparametric “trim-and-fill” method was used to determine the stability if publication bias was found.^[9]^ Generally, a 2-sided *P* value less than 0.05 was considered statistically significant, and we analyzed data with Review Manager 5.3.3 (Nordic Cochrane Centre, Rigshospitalet, Denmark) and STATA (Version 12.0 )(Stata Corp. LP, Lakeway Drive College Station, Texas, USA).

## 3 Results

**Figure 1:**
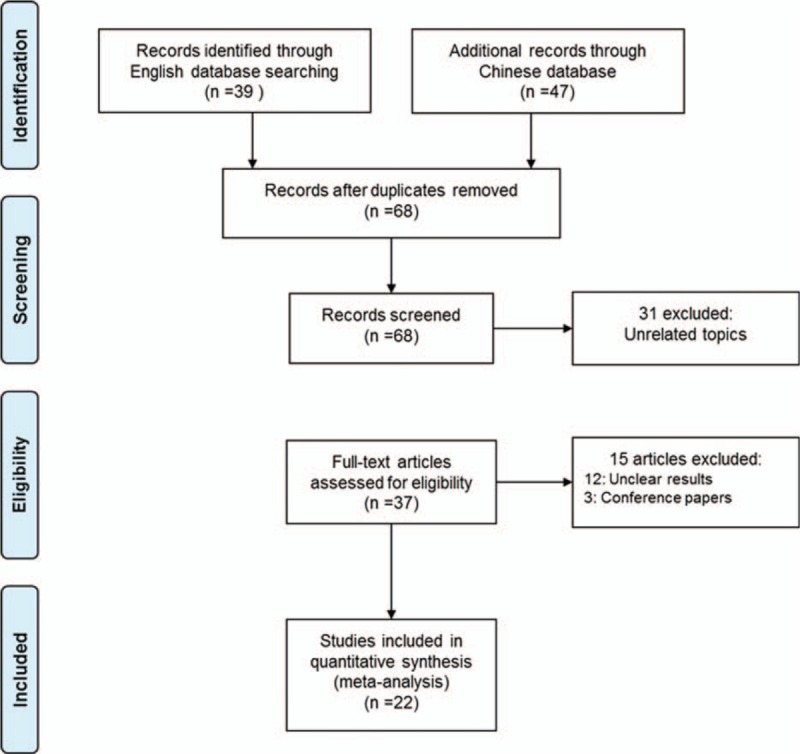
Flow diagram of the literature search and trials selection process (PRISMA Flowchart). PRISMA = Preferred Reporting Items for Systematic Reviews and Meta-analyses.

Our research returned 68 results after removing duplicates, from which we collected 22 trials in our meta-analysis^[10–31]^ (Fig. 1). All the studies recruited type 2 diabetes patients (Table 1 ). Two articles did not refer to the age of patients^[10,11]^; the subjects’ population age was over 35 years in the rest of the studies. In 5 studies, the average age (or median age) of the subjects was over 60 years old.^[12–16]^ Two studies were conducted in Spain and Germany^[12,13]^ and the rest were conducted in China. Three studies returned a Jadad Score of 3 or greater,^[12,13,17]^ all others were less than 3. However, the research conclusion is objectively based on blood index and the low design quality has little effect on our final conclusion.

**Table 1:**
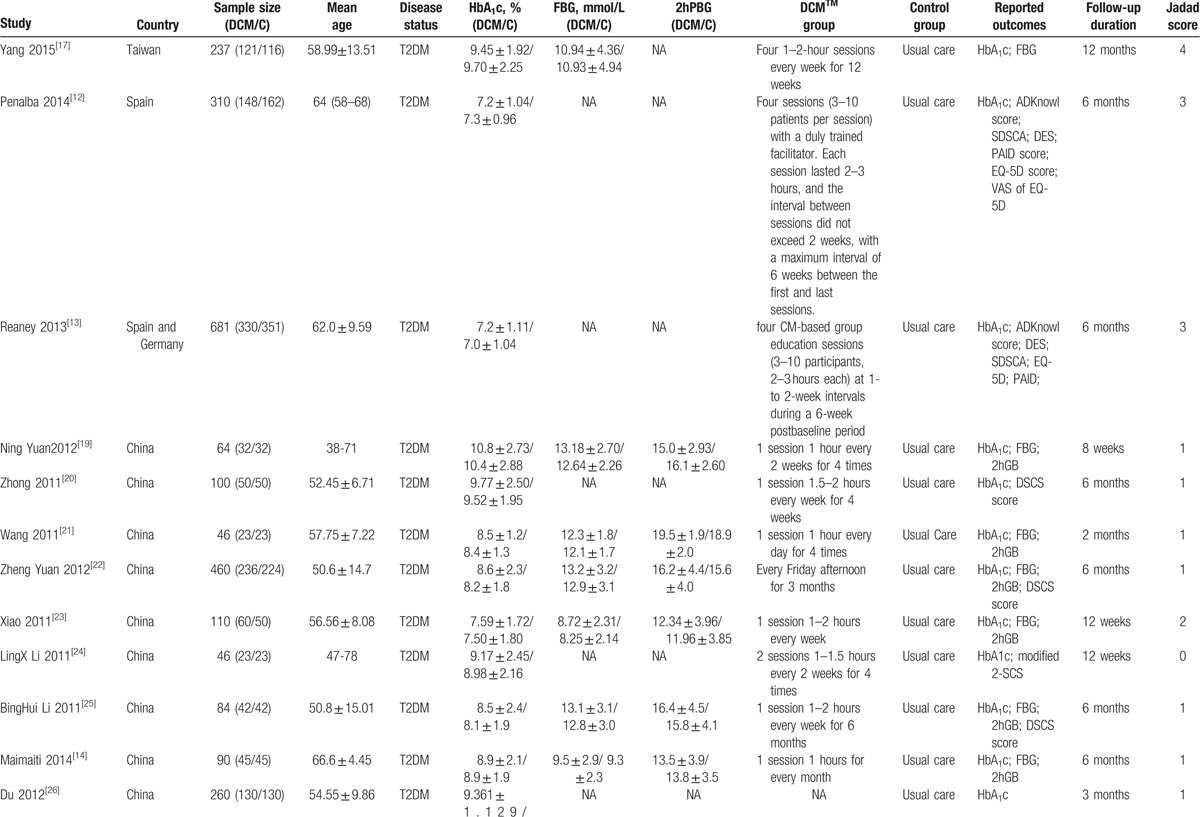
Baseline characteristics.

**Table 1 (Continued):**
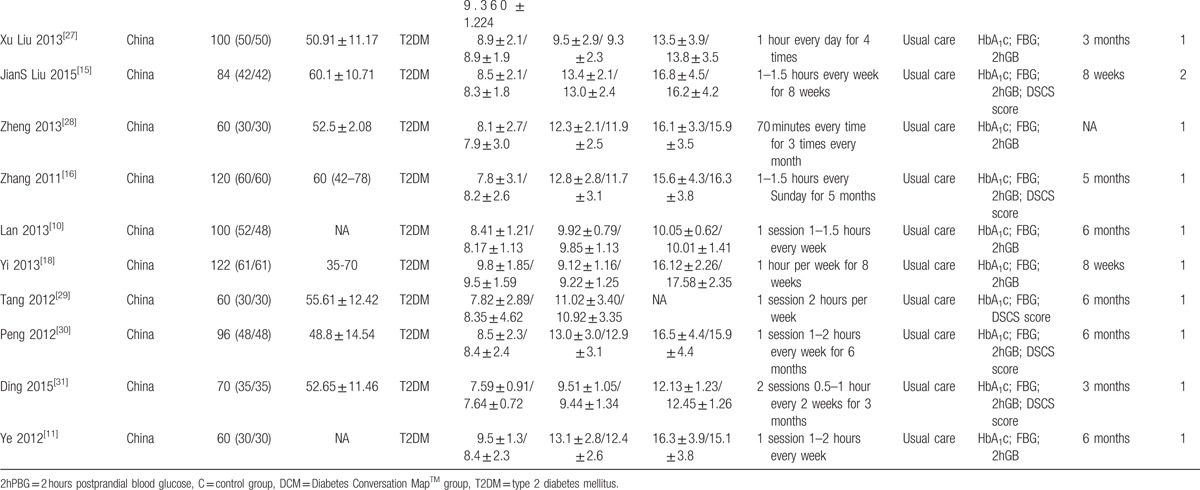
Baseline characteristics.

**Figure 2:**
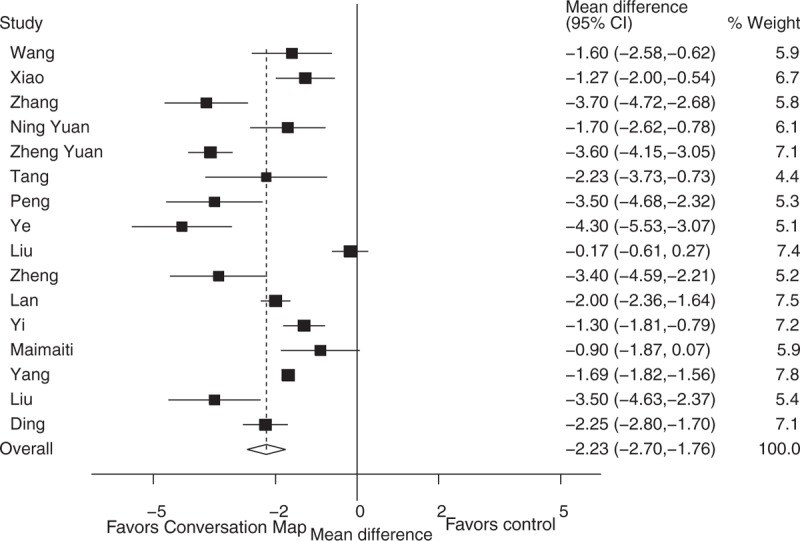
Effect of conversation map on fasting blood-glucose levels.

The FBG results show “Conversation Map” was significantly associated with the reduction of fasting blood-glucose levels when compared to control groups in type 2 diabetes patients (WMD: −2.23, 95% CI: −2.70 to −1.76, *P* < 0.001). However, there was heterogeneity among the studies (I^2^ = 90.7%, *P* < 0.001) (Fig. 2). Using subgroup analysis, we found that the heterogeneity was not significantly reduced when distinguished between the country (China or Europe), patients’ ages (<60 years old or ≥60 years old) and the education intervention time (<12 weeks or ≥12 weeks) (Table 2). The heterogeneity did not affect the overall results on the basis of country due to all of studies conducted in China. Similarly, conversation map education could significantly reduce FBG in patients less than 60 years old (WMD: −2.13, 95% CI: −2.83 to −1.43, *P* < 0.001) and in patients older than 60 years (WMD: −2.69, 95% CI: −4.52 to −0.85, *P* = 0.004) (Table 2). And the intervention time subgroup analysis results also showed that conversation map education could significantly reduce the levels of FBG within less than 12 weeks intervention (WMD: −1.94, 95% CI: −2.80 to −1.08, *P* < 0.001) and longer than 12 weeks (WMD: −2.06, 95% CI: −2.68 to −1.44, *P* < 0.001) (Table 2).

**Table 2:**
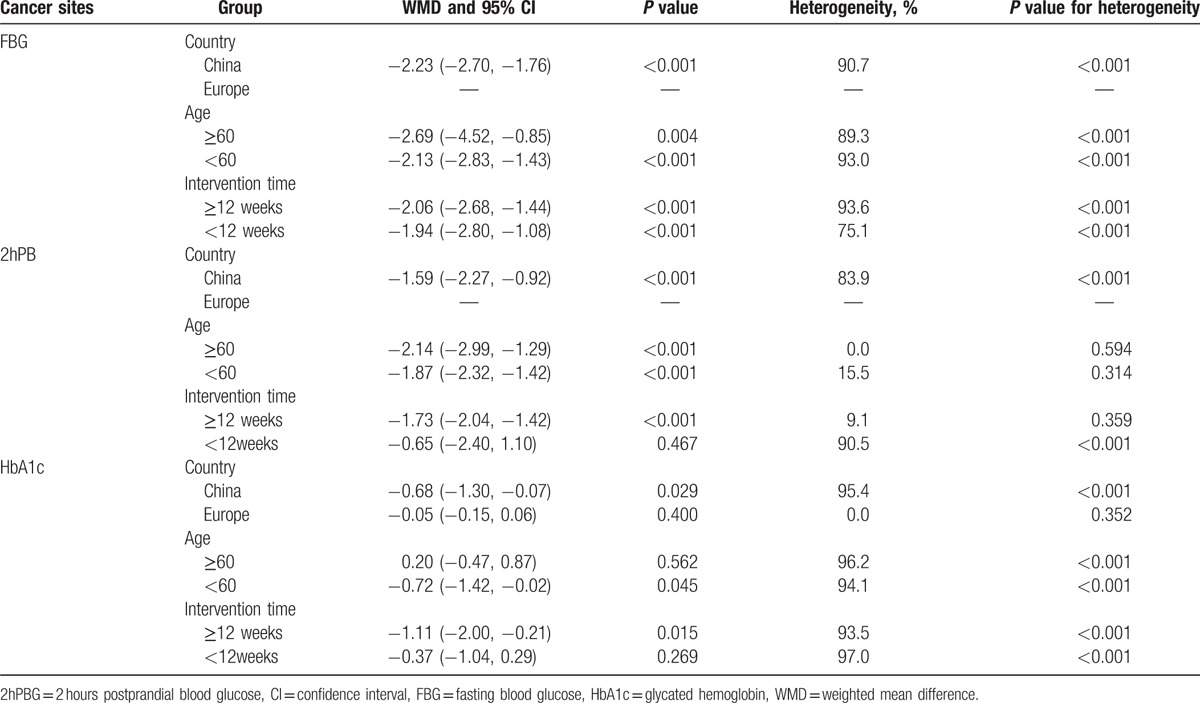
Subgroup analysis of effect of Conversation Map on type 2 diabetes.

**Figure 3:**
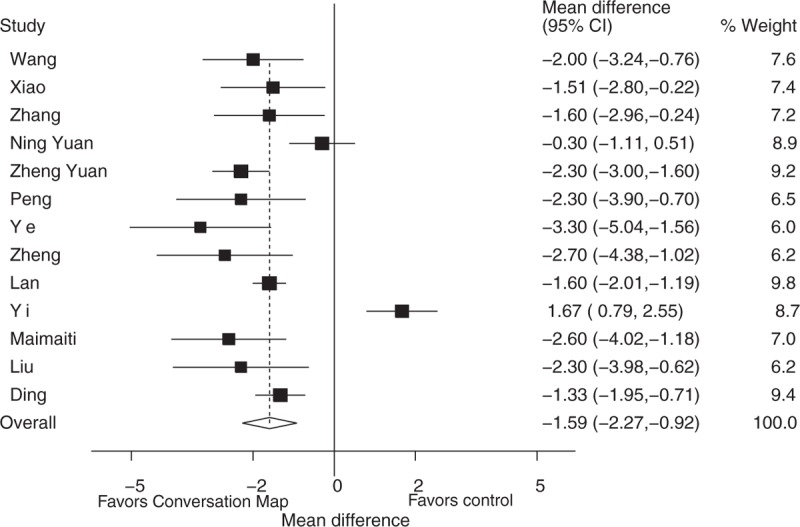
Effect of conversation map on 2-hour postprandial blood glucose levels.

The 2 hours postprandial blood glucose (2hPBG) results indicate that “Conversation Map” was significantly associated with the reduction of 2-hour postprandial blood glucose levels when compared to the control groups (WMD: −1.59, 95% CI: −2.27 to −0.92, *P* < 0.001). However, substantial heterogeneity among the studies was detected (I^2^ = 83.9%, *P* < 0.001) (Fig. 3). Using subgroup analysis on the basis of country was similar with overall analysis due to all of studies conducted in China. Furthermore, we found that the patients’ age was a source of heterogeneity (<60, I^2^ = 15.5%, *P* = 0.314; > = 60, I^2^ = 0%, *P* = 0.594). Finally, we found that there were no differences between the “Conversation Map” group and the control group when the intervention time was less than 12 weeks (WMD: −0.65, 95% CI: −2.40 to 1.10, *P* = 0.467) (Table 2).

**Figure 4:**
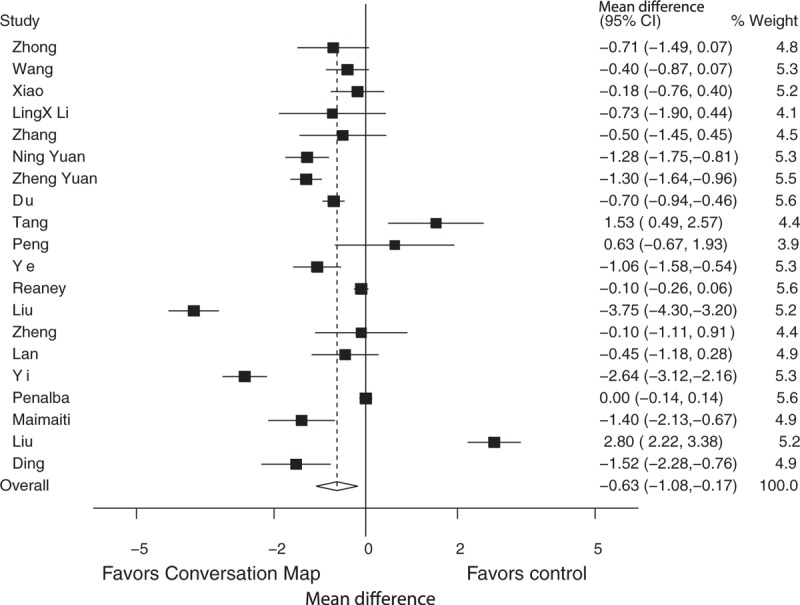
Effect of conversation map on Hemoglobin A1C levels.

The HbA1c results showed that “Conversation Map” was significantly associated with the reduction of the Hemoglobin A1C levels when compared to the control groups (WMD: −0.63, 95% CI: −1.08 to −0.17, *P* < 0.001), but there was heterogeneity found among the studies (I^2^ = 96.0%, *P* < 0.001) (Fig. 4). Using subgroup analysis, we found that country, the patients’ ages and the intervention times were not a source of heterogeneity. In our analysis, there was no significant differences between the groups when the study conducted in Europe (WMD: −0.05, 95% CI: −0.15 to 0.06, *P* = 0.400), the patient's age was over 60 years (WMD: 0.20, 95% CI: −0.47 to 0.87, *P* = 0.562), and the intervention time less than 12 weeks (WMD: −0.37, 95% CI: −1.04 to 0.29, *P* = 0.269) (Table 2).

**Figure 5:**
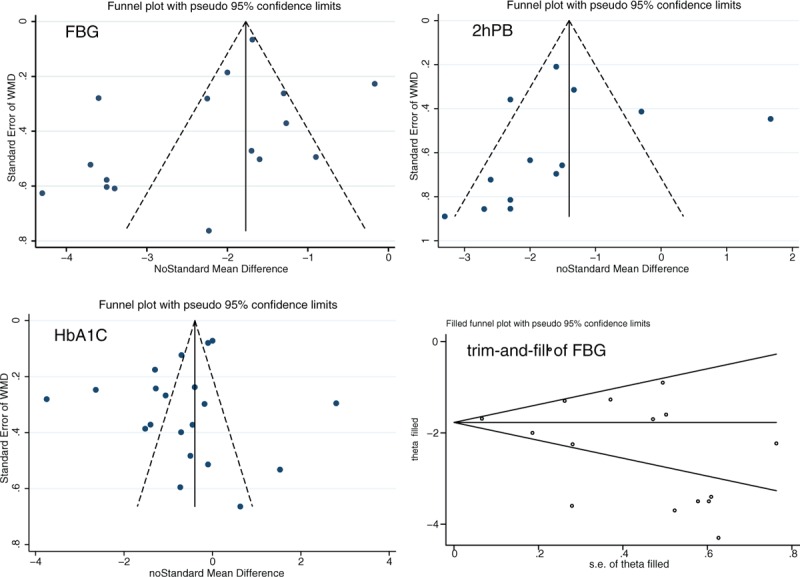
Funnel plot for FBG, 2hPBG, and HbA1c. 2hPBG = 2 hours postprandial blood glucose, FBG = fasting blood glucose, HbA1c = glycated hemoglobin.

The publication bias analysis indicated no existence of bias, except in FBG results (Begg's test, *P* = 0.053) (Fig. 5). After the “trim-and-fill” method^[9]^ correction, the results did not change.

## 4 Discussion

In this study, we analyzed the effectiveness of “Conversation Map” for diabetes patients. The study included information from 22 articles with 3360 patients. In the results, “Conversation Map” education was found to be significantly associated with the reduction of the FBG, 2hPBG, and HbA1C, indicating that this health education method might have an influence in controlling the blood glucose levels. “Conversation Map,” as a new form of diabetes education, could improve the patient's disease knowledge, improve their quality of life, and reduce consumption of medical resources in theory. However, the effectiveness of this education model greatly depends on the patient's compliance and education level.

Generally, “Conversation Map” can help change the patients’ living habits and to improve glycemic index. In long term, this method can reduce the diabetes complications, reduce disability rate and mortality, and reduce the consumption of social medical resources.^[32]^ In this study, we analyzed the heterogeneity of the studies using subgroup analysis of country, the patients’ age and intervention time for the following reasons. First, the patient's age can have implications on their cognitive ability and compliance; generally, older patients have lower cognitive ability and poor compliance.^[12,15,16]^ In 2hPBG results, age was a source of heterogeneity, but the results were robust. However, for the HbA1C results, there were more significant results in patients younger than 60 years old. Education intervention time is another important influential factor; shorter intervention time may not achieve desired results.^[18]^ For HbA1c levels, there was no significant difference between groups. In addition, the meta-regression analysis showed no significant correlation between the intervention time and the blood glucose index. Based on the results of this study, we found that 12 weeks of intervention time can produce patients’ interest of health education and can well control the patients’ blood glucose. In our study, the baseline hemoglobin A1c levels ranged 7.2% to 10.8 for the Conversation Map group and 7.0% to 10.4% for the control group, and the mean changes of hemoglobin A1c in Conversation Map group ranged from −6.9 to 0.1, and ranged from −6.8 to 0.1 in the control group. Minor changes were observed in individual studies, but the summary results indicated that the Conversation Map was associated with reduced levels of hemoglobin A1c by 0.63%. Although the size of heterogeneity was not minimized according to the subgroup analysis, we noted that Conversation Map significantly reduced the levels of hemoglobin A1c in patients with a mean age of less than 60 years, whereas no impact observed in patients with a mean age of greater than 60 years. This is due to lower cognitive ability and poor compliance of the older patients. Furthermore, subgroup analysis on the basis of country was performed, and we noted 2 studies^[12,13]^ conducted in Europe just reported hemoglobin A1c, whereas FBG and 2hPBG were not available. Although there was no significant difference between groups for hemoglobin A1c if the study conducted in Europe, whereas the result might variable due to smaller cohort included in this subset. Finally, although education levels might play an important role for intervention effect; however, the data about education levels in patients were available in few studies; we therefore did not provide the results in specific subpopulations with different education levels.

In this review, objective indicators, such as FBG, 2hPBG, and HbA1c, were used to reflect the patients’ blood glucose control in short and medium term. The subgroup analysis showed the study conducted in Europe, insufficient intervention time (<12 weeks) or the patients are too old (≥60) and hence the Conversation Map education may not achieve the desired effect. The reason for intervention effects according to country have already stated above. Furthermore, these results indicated that the education time should be longer than 12 weeks, and pay more attention to make patient understand when patient's age was over 60, if necessary intervention time and frequency should be increased.

To the best of the author's knowledge, this is the first systematic review of “Conversation Map” health education interventions for blood glucose index in diabetes patients, and no relative meta-analysis existed before. However, this research had several limitations. First, we did not have specific individual data for all the trials, thus our statistical analysis could only be performed at the study level. Second, although subgroup analysis was used, there was still heterogeneity presented. Third, the design risks of included studies were relatively high. We suggest a unification of health education methods and patient selection methods with a well-designed randomized double-blind placebo controlled trials to find a more reliable conclusion.

## 5 Conclusion

Conversation Map is an effective health education tool for type 2 diabetes, and could significantly reduce patients’ blood glucose related index. Further health education tools are needed to prevent and delay the development of type 2 diabetes mellitus in future.

## References

[1] Chinenye S Young EE Diabetes conversation map in Nigeria: a new socioeducational tool in diabetes care. *Indian J Endocrinol Metab* 2013; 17:1009–1011. 24381876 10.4103/2230-8210.122613 PMC3872677

[2] Panic N Leoncini E de Belvis G Evaluation of the endorsement of the preferred reporting items for systematic reviews and meta-analysis (PRISMA) statement on the quality of published systematic review and meta-analyses. *PLoS One* 2013; 8:e83138. 24386151 10.1371/journal.pone.0083138 PMC3873291

[3] Clark HD Wells GA Huet C Assessing the quality of randomized trials: reliability of the Jadad scale. *Control Clin Trials* 1999; 20:448–452. 10503804 10.1016/s0197-2456(99)00026-4

[4] Higgins JP Thompson SG Deeks JJ Measuring inconsistency in meta-analyses. *BMJ* 2003; 327:557–560. 12958120 10.1136/bmj.327.7414.557 PMC192859

[5] Xu S Liu H Xie Y Effect of mesenchymal stromal cells for articular cartilage degeneration treatment: a meta-analysis. *Cytotherapy* 2015; 17:1342–1352. 26122717 10.1016/j.jcyt.2015.05.005

[6] Begg CB Mazumdar M Operating characteristics of a rank correlation test for publication bias. *Biometrics* 1994; 50:1088–1101. 7786990

[7] Egger M Davey Smith G Schneider M Bias in meta-analysis detected by a simple, graphical test. *BMJ* 1997; 315:629–634. 9310563 10.1136/bmj.315.7109.629 PMC2127453

[8] Papageorgiou SN Dimitraki D Coolidge T Publication bias & small-study effects in pediatric dentistry meta-analyses. *J Evid Based Dent Pract* 2015; 15:8–24. 25666576 10.1016/j.jebdp.2014.09.001

[9] Mavridis D Salanti G How to assess publication bias: funnel plot, trim-and-fill method and selection models. *Evid Based Ment Health* 2014; 17:30. 24477535 10.1136/eb-2013-101699 PMC13404685

[10] Lan WX Wang Y Guo XP Application evaluation of figure dialogue tool in patients with type 2 diabetes. *Medical Forum* 2013; 17:4507–4509.

[11] Ye JH Ren XS Application of “figure dialogue” health education tool in type 2 diabetes management. *Inner Mongol Journal of Traditional Chinese Medicine* 2012; 31:116–117.(in Chinese).

[12] Penalba M Moreno L Cobo A Impact of the <<Conversation Map>> tools on understanding of diabetes by Spanish patients with type 2 diabetes mellitus: a randomized, comparative study. *Endocrinol Nutr* 2014; 61:505–515. 25085598 10.1016/j.endonu.2014.06.001

[13] Reaney M Zorzo EG Golay A Impact of conversation map education tools versus regular care on diabetes-related knowledge of people with type 2 diabetes: a randomized, controlled study. *Diabetes Spectrum* 2013; 26:236–245.

[14] Maimaiti RYL Peng QJ Application of the conversation map tool in the health education of uygur elderly type 2 diabetic patients. *Chin J Nurs* 2014; 20:1671–1674.

[15] Liu JS Lin YJ Effects of diabetic health education pictures on blood sugar control and self-care ability of type2 diabetic patients. *Modern Clinical Nursing* 2015; 14:34–37.(in Chinese).

[16] Zhang JX Zhang YY Huang XC Application of “Dialogue pictures” interactive education in patients’ blood glucose control with type 2 diabetes. *Guide of China Medicine* 2011; 9:330–331.(in Chinese).

[17] Yang YS Wu YC Lu YL Adherence to self-care behavior and glycemic effects using structured education. *J Diabetes Investig* 2015; 6:662–669. 10.1111/jdi.12343 PMC4627543 26543540

[18] Yi FS Wu SL Shen LL Clinical study of diabetic pictures dialogue tool for blood glucose control in patients with type 2 Diabetes Mellitus. *World Health Digest Medical Periodical* 2013; 10:181–182.

[19] Yuan N He MH Yang WX Clinic effect study of Conversation tool on glucose control for type 2 diabetes patients. *J Pract Diabetol* 2012; 8:37–38.

[20] Zhong WR Zeng RY Wang YE Influence of conversation education on self-management in diabetes mellitus patients. *J Pract Diabetol* 2011; 7:18–19.

[21] Wang XS Shi HZ Deng Q Influence of “Figure Dialogue” diabetes education tool application on blood glucose control for first visit type 2 diabetes mellitus patients. *Clin J Diabetes World* 2011; 5:262–263.

[22] Yuan Z Hu XH Chai H Effect evaluation of “diabetes figure dialogue” tool application in health education on type 2 diabetes patients. *Chin J Prac Nurs* 2012; 28:93–94.

[23] Xiao DM Zhang XQ Zhang YW Effect of glycemia and blood-lipid in patients with type 2 diabetes educated by diabetes conversation map. *Chin J Mod Nurs* 2011; 17:405–407.

[24] Li LX Zhou X Wang N Application of figure conversation toolTM in diabetes education for type 2 diabetes patients. *Chin J Mod Nurs* 2011; 17:654–655.

[25] Li BH Zhang C Zhu WD Effect of interactive education with conversation map on the control of blood glucose in patients with type 2 diabetes. *Chin J Nurs* 2011; 46:48–50.

[26] Du XX Li SL Wang XY Discussion on figure dialogue tool for diabetes patients treated with insulin. *China Medicine and Pharmacy* 2012; 2:199–200.(in Chinese).

[27] Liu X Li H Xu XF Application of dialogue education mode in health education on type 2 diabetes mellitus patients. *Med Inform* 2013; 26:281.

[28] Zheng GY Figure dialogue” interactive education in glycemic control in patients with type 2 diabetes. *Mod Hosp* 2013; 13:151–152.

[29] Tang YY Li T Zhang Y The effect of education of conversation with picture for behavior change in type 2 diabetic patients. *Sichuan Med* 2013; 33:730–732.

[30] Peng XL Li XH The effect evaluation of “Figure dialogue” interactive education on type 2 diabetes patients. *Pract J Cardiac Cerebral Pneumal Vas Dis* 2012; 20:85–86.

[31] Ding DC Liu L Ye FL Application of “Figure dialogue” interactive education model in patients with type 2 diabetes mellitus and depression. *J Clin Nurs* 2015; 14:40–42.

[32] Crawford P Wiltz S Participation in the journey to life conversation map improves control of hypertension, diabetes, and hypercholesterolemia. *J Am Board Fam Med* 2015; 28:767–771. 26546652 10.3122/jabfm.2015.06.140142

